# Effect of Grain Boundary Characteristics on Mechanical Properties and Irradiation Response in 3C-SiC: A Molecular Dynamics Simulation Study

**DOI:** 10.3390/ma18153545

**Published:** 2025-07-29

**Authors:** Wenying Liu, Fugen Deng, Jiajie Yu, Lin Chen, Yuyang Zhou, Yulu Zhou, Yifang Ouyang

**Affiliations:** 1 State Key Laboratory of Featured Metal Materials and Life-Cycle Safety for Composite Structures, School of Physical Science and Technology, Guangxi University, Nanning 530004, China; 2 Guangxi Key Laboratory for Relativistic Astrophysics, School of Physical Science and Technology, Guangxi University, Nanning 530004, China

**Keywords:** silicon carbide, symmetric tilt grain boundaries, mechanical properties, molecular dynamics, cascade collision, grain boundary characteristics

## Abstract

Molecular dynamics (MD) simulations have been performed on the energetics, mechanical properties, and irradiation response of seventy-three 3C-SiC symmetric tilt grain boundaries (STGBs) with three tilt axes (<100>, <110> and <111>). The effect of GB characteristics on the STGB properties has been investigated. The GB energy is positively and linearly correlated with the excess volume, but the linearity in SiC is not as good as in metals, which stems from the inhomogeneous structural relaxation near GBs induced by orientation-sensitive covalent bonding. For <110>STGBs, the shear strength exhibits symmetry with respect to the misorientation angle of 90°, which is consistent with ab initio calculations for Al in similar shear orientations. Cascades are performed with 8 keV silicon as the primary knock-on atom (PKA). No direct correlation is found between the sink efficiency of GBs for defects and GB characteristics, which comes from the complexity of the diatomic system during the recovery phase. For GBs with smaller values of Σ, the GBs exhibit a weaker blocking effect on the penetration of irradiated defects, resulting in a lower number of defects in GBs and a higher number of total surviving defects. In particular, it is seen that the percentage decrease in tensile strength after irradiation is positively correlated with the Σ value. Taken together, these results help to elucidate the impact of GB behavior on the mechanical properties of as well as the primary irradiation damage in SiC and provide a reference for creating improved materials through GB engineering.

## 1. Introduction

Silicon carbide (SiC) is one of the most attractive ceramics in the field of the nuclear [1,2,3] and electronics [4,5] industries because of its good irradiation resistance, high thermal conductivity, and excellent mechanical strength. For example, in high temperature gas-cooled reactors, the SiC layer is a critical layer of the tristructural isotropic (TRISO)-coated fuel particles, which serves as a pressure vessel for the fuel and should have sufficient strength to withstand mechanical stress during normal and extreme conditions.

Grain boundaries (GBs) play a dominant role in the mechanical properties of nanocrystalline SiC. Generally, GBs exhibit weaker mechanical properties than grain interiors, so crack nucleation is more inclined to start near GBs, especially those nearly perpendicular to the loading axis [6]. Some experiments have explored the effect of grain refinement on elastic modulus, hardness, and residual stress of SiC [7,8,9]. The GB structure has a significant influence on corrosion [10,11], diffusion of element [12], ductility [13,14], crack propagation [15], etc. In SiC, the presence of high-angle grain boundaries (HAGBs) increases the corrosion rate and diffusion coefficient due to their disordered structures and high energies [10,12]. Incoherent GBs were found to be more vulnerable to oxidation than single crystals [11]. GB engineering in austenitic stainless steels leads to a higher fraction of coincident lattice site (CSL) GBs, which enhances elongation in tensile tests [13,14]. Therefore, it is necessary to examine the connection between GB characteristics and the mechanical response of the material.

Molecular dynamics (MD) methods provide atomic-scale insights into the dynamics of mechanical deformation and defect evolution. In order to systematically investigate the feature of individual GB, bicrystalline structures are often constructed in MD simulations. The main concerns include interfacial energy [16,17,18,19,20,21,22] and mechanical response under tensile loading [20,23,24,25,26] of bicrystalline systems. The tensile strength of bicrystal is typically lower than that of the bulk, but the effect of GB characteristics on strength has remained unclear. By analyzing metastable Σ5<100>{120} and Σ9<110>{122} GBs using machine learning techniques, Guziewski et al. found that the GB excess free volume primarily dominates the tensile strength [24]. On the other hand, some studies suggested that the tensile strength correlates with the misorientation angle of GBs. A study of seven asymmetric <100> tilt GBs showed that relatively low misorientation GBs (0° ≤ *θ* < 40°) have higher tensile strength than those with relatively high GB misorientation (40° ≤ *θ* < 62°) [26]. A similar trend in tensile strength was observed for four <100> symmetric tilt grain boundaries (STGBs) in Fe [27], while the results for forty-two <100> STGBs in W exhibited the opposite trend [28]. Studies of Cu STGBs showed that GBs with the “zigzag” or “fishbone” shape can effectively prevent GB distortion, thereby improving the GBs’ stability and increasing the yield stress of the material [28]. Regarding the shear response, ceramic bicrystals generally do not exhibit stick–slip or GB migration, which is common in metals. Bringuier et al. [19] reported for the first time a shear-induced stick–slip behavior in SiC Σ365<100>4.24° STGB, which has not been previously reported in materials that exhibit purely covalent bonding characteristics. Previous research explored the connection between the GB shear strength and eight common GB descriptors in Al twist GBs, but no relationship was found [29]. In ab initio calculations for five STGBs in Al, Pang et al. [30] found that the shear strength is not directly related to the Σ value or the GB energy. Instead, it exhibits a relation with the misorientation angle, with extreme values occurring when the misorientation angle is close to 90°. In a recent study, Shao et al. [31] compared the shock response of four SiC STGBs and found that the response mechanism varied with the tilt angle.

The harsh irradiation environments of nuclear systems pose challenges to SiC. The bombardment of neutrons and ion fluxes results in the formation of radiation defects such as point defects and clusters. The GBs act as sinks for radiation-induced defects, improving radiation tolerance due to enhanced defect recovery. Experiments showed that the type of GB influences its sink characteristics for defects [15,32,33]. In SiC and other materials, the segregation ability of interstitials was found to be correlated with the excess volume [34,35,36], GB energy [37,38], twist axis [39], or misorientation angle [37], whereas the segregation ability of vacancies may be related to the Σ value [40] or GB energy [41]. Defects arising from irradiation induce local stress at GBs, which can cause changes in the mechanical behavior of the material [42]. Cui et al. [43] compared the solution and diffusion behaviors of hydrogen in SiC. It is also found that the temperature dependence of radiation-induced segregation in ceramics is different from that shown in metallic systems [44]. Calculations showed that the tensile strength of irradiated-SiC significantly deteriorates, and the deterioration is significantly higher in bicrystals compared to the single crystalline structure [20,45,46]. However, few studies have addressed the effect of GB characteristics on changes in mechanical properties after irradiation. Studies of eight <100> and <110>STGBs in Fe showed that the loss in tensile strength after irradiation is higher for Σ3 GBs than for the other GBs, and materials with low angle GBs would have good irradiation resistance [47]. Calculations of more than ten STGBs in Zr suggested that the tensile strength after irradiation correlates only with the misorientation angle [48].

A comprehensive knowledge of the mechanical behavior and irradiation response of SiC GBs is needed. Most of the previous simulations have focused on the interfacial energy of <100> and <110>STGBs, while several works have been concerned with the tensile mechanical response of <100> STGBs. Regarding the shear response and irradiation behavior, previous studies are calculated based on a few specific GBs, and the applicability of the findings is worth further exploration. Furthermore, previous efforts have examined the connection between GB characteristics and material properties in metallic systems such as Fe and Al, while few studies have been conducted in SiC. In this study, MD simulations have been performed on the energetics, mechanical properties, and irradiation response of seventy-three 3C-SiC STGBs with three tilt axes (<100>, <110>, and <111>). The effect of GB characteristics on the STGB properties has been focused on. The results are expected to provide useful information for understanding the behavior of GBs in SiC and provide a reference for creating improved materials via GB engineering.

## 2. Simulation Method

Simulations were performed using the LAMMPS program (Large-Scale Atomic/Molecular Massively Parallel Simulator [49], Version: 27Oct2021), whereas the visualization and analysis were accomplished using the Open Visualization Tool (OVITO, Version: ovito-basic-3.7.2-win64) [50]. The Tersoff bond-order interatomic potential [51] modified by Kohler [16] was used to describe the interactions in SiC. Kohler adjusted the Si–Si cut-off from 3.0 Å to 2.85 Å. The reason is that in highly distorted configurations of SiC, such as dislocations and GBs, a cut-off of 3 Å leads to interactions between the second nearest neighbor atoms of Si and an unphysical increase in the energy. This modified Tersoff potential has been proven to reproduce the structure of SiC tilt GBs accurately and provides results more consistent with ab initio calculations compared to the original Tersoff potential. It has been used to investigate the behavior of SiC GBs [17,21,26]. In irradiation simulations, the Ziegler–Biersack–Littmark (ZBL) potential [52], a universal repulsive potential known to account well for high-energy collisions at close separations, was employed for short-range interactions. The ZBL portion of the Tersoff/ZBL potential [53] contains the Coulomb repulsive term and the ZBL universal screening function term. The potential is widely used in cascade simulation of SiC [20,54].

### 2.1. Generation of GBs

The SiC STGB models with varying misorientation angles were constructed via the CSL method, with Σ representing the CSL index. A total of seventy-three GB orientation structures were considered, including <100>, <110>, and <111> STGB systems. In each simulation cell, the *z* direction was parallel to the tilt rotation axis, while the *y* direction was perpendicular to the GB plane.

For the calculations of the energies, the total number of atoms in the bicrystal system was approximately 10,000. In particular, the height of the bicrystal *L_y_* should be large enough to avoid interactions between the two GB planes under periodic boundary conditions along the *y* direction during the relaxation process. Depending on the period lengths of different crystal orientations, the value of *L_y_* varied across different models, ranging from 100 Å to 180 Å. For the calculations of mechanical properties, the total number of atoms in the bicrystal system was approximately 30,000. The *y* direction was about twice as long as the other two directions. For the irradiation simulation, the system was expanded to include approximately 1,000,000 atoms to guarantee that the irradiation defects would not penetrate the boundaries of the substrate. The value of *L_y_* ranged from 285 Å to 360 Å. The size of each system can be found in Tables S1–S9.

The systems were optimized by the method outlined by Wojdyr et al. [17]. In this process, the upper grain was displaced in increments of 0.2 Å within both interface plane directions. Then, some possible configurations with certain misorientation angles were obtained. Each configuration was then subjected to an annealing step in which the temperature of the system was first increased to 3000 K in 5000 steps and slowly cooled to 100 K in 20,000 steps. The adopted timestep was 1 fs. Every 5000 timesteps a snapshot of the atomic configurations was recorded and then minimized with the conjugate gradient (CG) algorithm. The structure with the lowest GB energy was considered as the optimized one for the given misorientation angle.

### 2.2. Energetics of GBs

The GB energy was calculated by taking the difference between the energy of the bicrystal and that of an ideal crystal with the same number of atoms and dividing by the area of the GB plane [22]

(1) $$\gamma_{\mathrm{GB}}=\frac{E_{\mathrm{tot}}-NE_{\mathrm{SiC}}}{2A_{\mathrm{GB}}}$$

where *E*_tot_ and *N* are the total energy and the number of atoms of the bicrystal system, respectively, and *E*_SiC_ is the per-atom cohesive energy of perfect SiC. *A*_GB_ is the area of the GB plane, which is counted twice due to the use of periodic boundary conditions in the direction perpendicular to the GB plane. The GBs with smaller energies are energetically favorable structures. We note that for non-stoichiometric GBs, the difference in chemical potentials of the two atoms should also be considered [16,21]. The focus of the work presented here is on stoichiometric GBs.

### 2.3. Tensile and Shear Deformation

**Figure 1:**
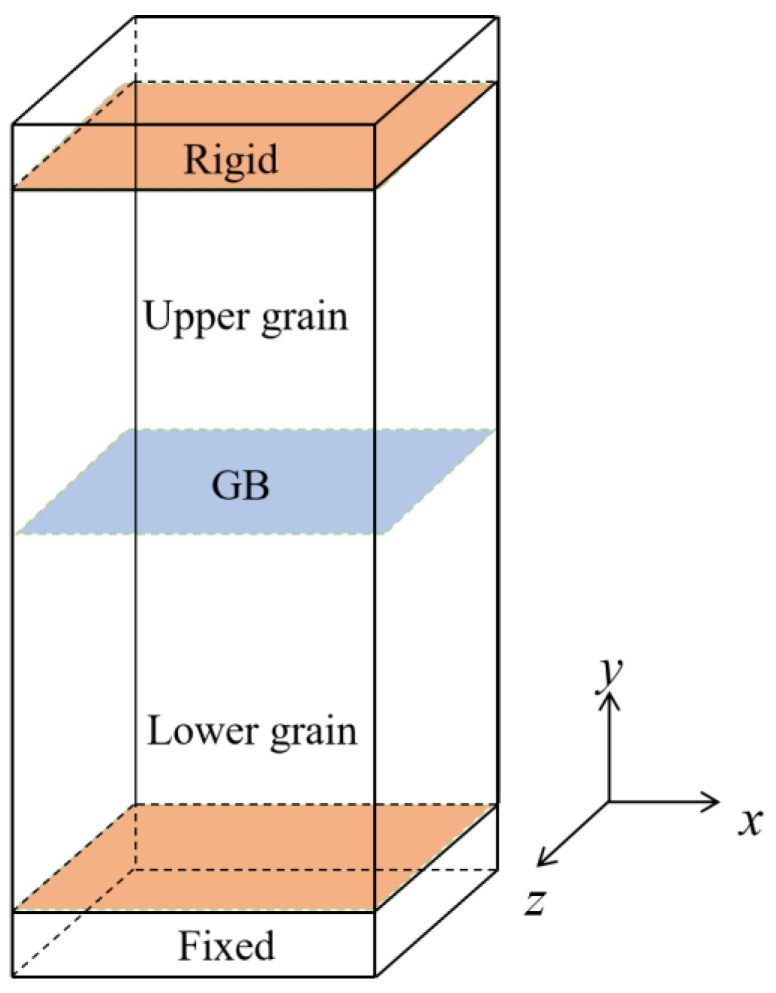
Schematic of SiC bicrystal model.

Prior to the application of strain, the bicrystals were subjected to thermal equilibration. The temperature was first raised to the desired temperature (*T* = 300 K) and then maintained for 50 ps in the canonical ensemble (NVT). Then, the system was equilibrated for 50 ps at the desired temperature using the isothermal–isobaric ensemble (NPT) to ensure the pressure was set to zero. Periodic boundary conditions were applied in all three directions. During the tensile deformation, the periodic boundary condition was removed perpendicular to the GB plane. The top and bottom atoms, twice as thick as the cutoff radius for atomic interactions, were treated as rigid, while the other atoms were allowed to move freely. The strain-controlled tensile deformation was performed in the NPT ensemble by applying a constant velocity of 0.01 Å ps^−1^ in the positive direction of the *y* axis to the top rigid atoms, while the bottom rigid atoms were kept fixed. The velocity gradient was applied to the atoms within one-third of the upper grain. The strain rate applied in our MD simulations is similar to previous MD simulations. Although it is higher than the strain rate in experiments, it still provides details for understanding the mechanisms of material deformation. A timestep of 1.0 fs was utilized. The application of shear strain was conducted similarly to tensile strain. The difference was that the fixed velocity was set along the *x* direction (positive and negative *x* axis). The bicrystal structure is shown in Figure 1.

### 2.4. Irradiation Simulation

For the simulation of the damage caused by irradiation, a Si atom was randomly selected as the primary knock-on atom (PKA) from a position four lattice constants away from the GB plane in the lower grain. The initial kinetic energy of PKA was set as 8 keV, which is slightly smaller than the peak of the nuclear stopping power for Si in SiC [55], with the velocity directed along the positive *y* axis. The NPT ensemble was applied to relax the system at 300 K, followed by the application of the microcanonical ensemble (NVE) during the cascade process. After the irradiation, the system was annealed at the desired temperature and then subjected to either tensile or shear deformation, as described above. An adaptive time step, ranging from 10^−3^ fs to 1 fs, was employed during the cascade process to ensure that all the atoms do not move by more than 20% of the nearest neighbor distance in SiC at each step [56]. Each cascade process lasted 6.1 ps. Considering the stochastic nature of the irradiation process, ten simulations were performed with different random seeds for each GB configuration. The total number of cascade simulations was 730.

**Figure 2:**
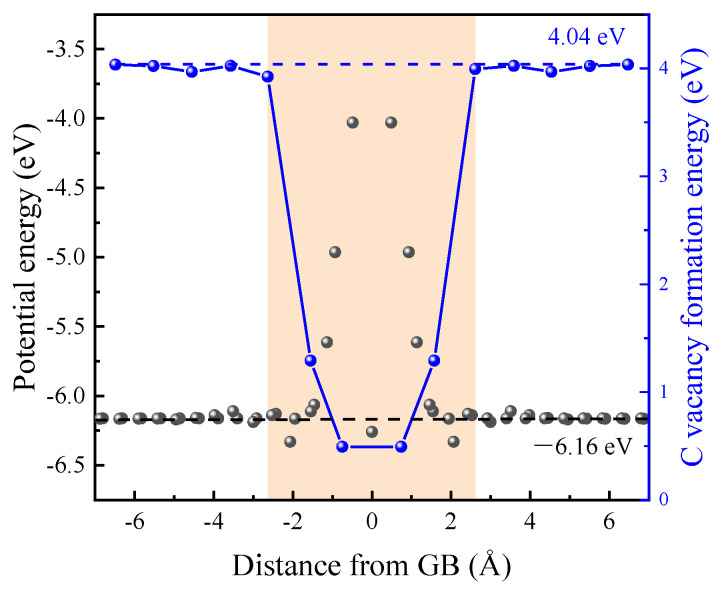
Average potential energy and C vacancy formation energy near Σ5<100>53.13**°** STGB. The average cohesive energy and C vacancy formation energy of bulk SiC are −6.16 eV and 4.04 eV, respectively. The GB region is represented by orange background.

The irradiation-induced point defects were identified using the Wigner–Seitz method. When studying the role of GB in the evolution of irradiation defects, it is essential to determine the region of GB. In an inclined GB structure, the potential energy near GB increases significantly due to the relative rotation of the two grains, which can serve as a criterion for determining the GB region. Taking Σ5<100>53.13° STGB as an example, the average potential energy in each layer is shown in Figure 2. The average cohesive energy of bulk SiC is −6.16 eV. Therefore, we defined the region where the potential energy exceeds the interval of (−6.16 ± 0.05) eV as the GB region. The variation in the C vacancy formation energy, defined as the energy cost of removing a C atom from the bicrystal and placing it in diamond, gives a similar range of GBs.

## 3. Results and Discussion

### 3.1. GB Energies

**Figure 3:**
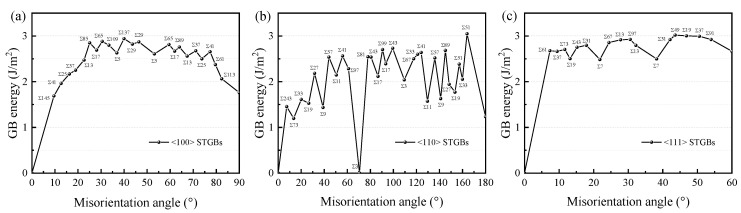
The energies of (**a**) <100> STGBs, (**b**) <110>STGBs, and (**c**) <111> STGBs as a function of the misorientation angle.

The GB energy of different misorientation angles was determined by the optimization scheme. The GB formation energies of twenty-four <100> STGBs, thirty-one <110>STGBs, and eighteen <100> STGBs are shown in Figure 3. The energies of some <100> and <110>STGBs have been calculated previously [16,17,19,20,21,22], and our results are consistent with most of them. In general, the smaller the value of Σ, the lower the GB energy, indicating higher thermodynamic stability and stronger cohesive bonding between the two grains. For <100> STGBs with low angles, the GB energy increases with the increasing misorientation angle. This trend is a typical characteristic of <100> STGBs across various materials and can be understood through the Read–Shockley dislocation model. For large-angle <100> STGBs, the distance between dislocations is small, and the interaction between dislocations causes the change in dislocation structure [17]. For <110>STGBs, one of the cusps in the energy curve corresponds to the Σ3<110>70.53° GB, which is coherently bounded and exhibits the lowest GB energy (~0 J m^−2^). Our results are consistent with the experimental observation that the largest fractions of CSL GBs (3 ≤ Σ ≤ 17) are the Σ3 GBs, followed by Σ9 GBs with 3–5% [10,12]. For <111> STGBs involving both C and Si atoms at the interface, the GB energy is relatively large.

**Figure 4:**
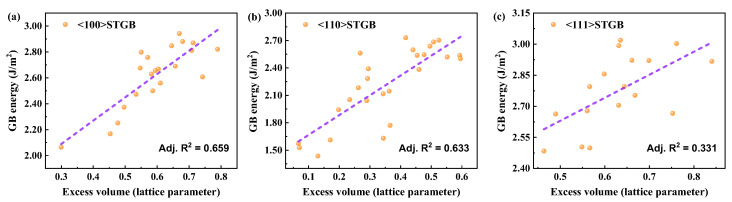
The GB energy as a function of the excess volume in (**a**) <100> STGBs, (**b**) <110>STGBs, and (**c**) <111> STGBs.

To accommodate the lattice mismatch, the two grains must expand locally at the GB. Consequently, the volume of the bicrystal system is larger than that of a perfect crystal composed of the same number of atoms. The expansion caused by GBs can be measured by excess volume, which can be represented by the equation in the form Δ*h* = (*V*_BC_ − *V*_SA_)/2*A*_GB_, where *V*_BC_ is the volume of the bicrystal, *N* is the number of atoms in the bicrystal, *V*_SA_ is the per-atom volume of perfect crystal, and *A*_GB_ is the interface area between two grains within the bicrystal system. A larger GB excess volume indicates greater lattice expansion near the interface, corresponding to a larger GB energy. A linear relationship between GB energy and excess volume has been observed in metals and alloys [57,58,59,60], where the proportionality coefficient depends on the specific type of metal. Guziewski et al. [24] found that the excess volume of GB is the most important macroscopic descriptor when predicting the energetics of GB in SiC through a machine-learning technique. This study shows that (Figure 4) a linear relationship can still be approximated between GB energy and excess volume in SiC. The slopes *β* for <100>, <110>, and <111> STGBs are found to be 1.80, 2.17, and 1.11 J/(m^2^*a*_0_), respectively (where *a*_0_ is the perfect lattice parameter of SiC lattice). However, the linearity is not as good as in metals or alloys, especially for the larger interfacial energies of <111> STGBs, as evidenced by the coefficients of determination (R^2^) between GB energy and excess volume. Covalent bonds exhibit higher sensitivity to orientation compared to metallic bonds; this leads to inhomogeneous structural relaxation near GB, which particularly weakens bonding interactions in the direction perpendicular to the GB plane.

### 3.2. Tensile Response of GBs

**Figure 5:**
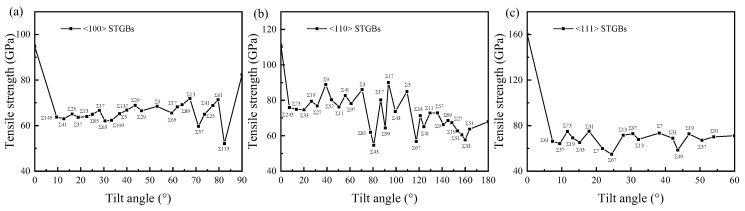
The tensile strength of (**a**) <100> STGBs, (**b**) <110>STGBs, and (**c**) <111> STGBs as a function of misorientation angle.

After the bicrystal is equilibrated at a temperature of *T* = 300 K using the NPT ensemble, uniaxial tensile strain is applied in the direction perpendicular to the GB plane to examine the stress–strain response of the bicrystal system. The tensile stress–strain curves of typical STGBs with <100>, <110>, and <111> tilt axes at 300 K are shown in Figure S1. At low strains, the tensile stress–strain relationship follows a linear Hooke’s law. As the applied strain gradually increases, the tensile stress reaches a peak value corresponding to the tensile strength. Fracture is always brittle, which demonstrates the properties of ceramic materials. Investigating the ideal strengths of crystals is an attractive problem because they represent the upper limit on the actual strength of crystalline materials. Figure 5 illustrates the tensile strength of STGBs in SiC as a function of misorientation angle. It is common for bicrystals to exhibit lower tensile strength compared with bulk SiC (misorientation angle = 0°). For <100> STGBs, our results align with those of Li et al. [20], who reported that the average strength of GBs is approximately 60% of the strength of bulk SiC, despite different atomic potentials being employed. While previous studies primarily focused on the tensile strength of <100> STGBs [20,25], this study finds that the tensile strength of <110>STGBs is higher. The tensile strength of <111>STGBs is similar to that of <100>STGBs.

In general, GBs with lower Σ values exhibit higher tensile strengths, which are closely associated with lower GB energies and smaller excess volumes. Conversely, GBs with higher Σ values are more susceptible to fracture under tensile loading due to the elongation and breakage of bonds caused by lattice expansion and mismatch. Machine learning studies on SiC indicated that the GB tensile strength depends primarily on the excess volume of GBs and, to a lesser extent, on the type of GB structure and the number of C-C bonds [23]. Han et al. found that the tensile strength decreases proportionally to the misorientation angle in their study of seven asymmetric <100> tilt GBs in SiC [26]. A similar trend was observed for four <100> STGBs in Fe [27]. However, studies on Cu [28] suggested that the yield strength of <100> STGB declines as the misorientation angle increases, which is believed to be related to the inherent resistance of certain GB structural units to the initial nucleation of the dislocations. In ceramic materials like SiC, where the energy required for dislocation initiation is considerably high, the tensile strength of GB is primarily determined by the excess volume of GB.

It is also found that some GBs do not satisfy this relationship. For example, the tensile strength of Σ33<110>159.95° GB is lower than that of Σ51<110>157.15° GB. This discrepancy arises because the structural optimization of the bicrystal system relies on the minimum potential energy principle. In some bicrystals, significant movement of the upper grain causes a loss of symmetry at the GB (Figure S2), so that their strength properties are no longer the same as those of other GBs within the local angle range.

### 3.3. Shear Response of GBs

The shear strain is applied in the *x* direction parallel to the GB plane to analyze the stress–strain response of the bicrystal system. The shear stress–strain curves of typical STGBs with <100>, <110>, and <111> tilt axes at 300 K are presented in Figure S3. The curves are linear at the initial stage, representing the elastic response, after which no stick–slip or GB migration behavior commonly seen in metallic bicrystals is observed [61,62,63]. The deformation of the SiC STGBs is primarily due to frictional drag at the GB. Bringuier et al. [19] reported for the first time a shear-induced stick–slip behavior in Σ365<110>4.24° STGB of SiC, which has not been reported in any material exhibiting purely covalent bonding characteristics. The stick–slip behavior may occur for low-angle GBs at low temperatures, whereas it is difficult for a much larger number of GBs with small Σ values.

**Figure 6:**
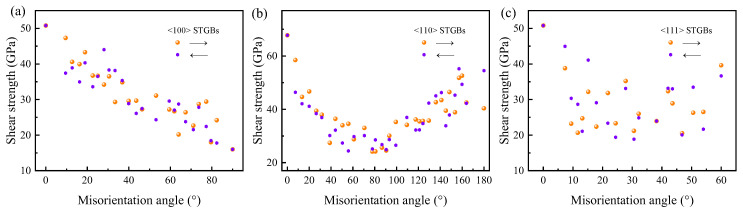
The relationship between the shear strength and the misorientation angle of STGBs at 300 K: (**a**) <100> STGBs, (**b**) <110>STGBs, and (**c**) <111> STGBs. In order to avoid the effect of the relative displacement between the two grains after structure optimization on the initial shear response, the shear load is respectively applied along the positive and negative *x* axis to the rigid region of the upper grain, as shown by the arrows.

As the strain increases, the bicrystal suddenly undergoes brittle failure. The shear strength is defined as the maximum shear stress obtained in the shear stress–strain curve. Previous studies have explored the relationship between shear strength and simplified descriptors of GB, such as CSL value, GB energy, and excess volume, but no comprehensive correlations were found [29]. This study found no clear correlation between the shear strength and either Σ value or GB energy. However, there is a relationship between shear strength and misorientation angle (see Figure 6). For <100> STGBs, shear strength demonstrated an approximately linear relationship with the misorientation angle. For <110>STGBs, shear strength exhibits symmetry with respect to the misorientation angle of 90°. As the misorientation angle closed to 90°, the shear strength decreases. Next, we will show that the normal stress near GBs plays an important role in shear strength variation.

**Figure 7:**
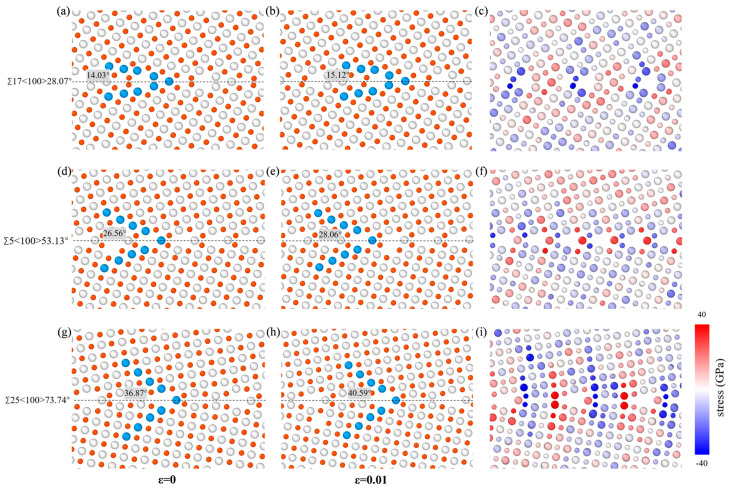
The variation in the angle between the upper grain and the GB plane of Σ17<100>28.07°, Σ5<100>53.13°, and Σ25<100>73.74° STGBs at the strain of (**a**,**d**,**g**) *ε* = 0; (**b**,**e**,**h**) *ε* = 0.01. The inset shows the angle between the upper grain and the GB plane, with the dashed line representing GB plane. (**c**), (**f**), and (**i**) correspond to the normal stress distribution of (**b**), (**e**) and (**h**), respectively. The grey and red balls represent Si and C atoms, respectively.

For <100>STGBs, Figure 7 takes Σ17<100>28.07°, Σ5<100>53.13°, and Σ25<100>73.74° as three typical STGBs to show how the angle between the upper grain and the GB plane varies, as well as the distribution of atomic normal stress, with the shear direction of the upper grain oriented to the right. As the shear strain increases from *ε* = 0 to *ε* = 0.01, the increments in the angle between the upper grain and the GB plane are 1.09°, 1.50°, and 3.72°, respectively. This demonstrates that <100> STGBs with larger misorientation angles are more susceptible to deformation under shear loading. Figure 7c,f,i show the normal stress distribution at *ε* = 0.01. The GBs exhibit alternating compressive and tensile regions [64,65]. As the misorientation angle increases, the non-uniformity of stress distribution near the GB becomes more pronounced, resulting in decreased local mechanical stability. Additionally, the average normal stress in the GB region slightly decreases with the increase in the orientation angle. A smaller normal stress indicates larger interatomic distances in the direction perpendicular to the interface, which reduces the number of bonds across the GB and decreases the frictional drag, thus exhibiting a smaller shear strength.

**Figure 8:**
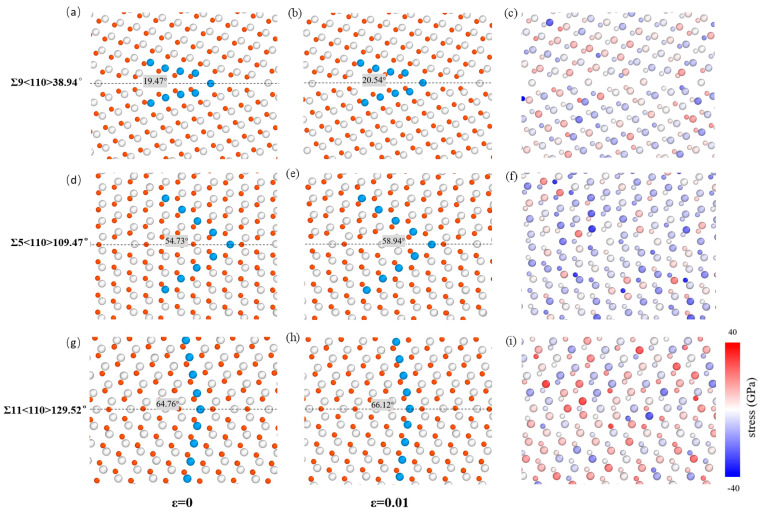
The variation in the angle between the upper grain and the GB plane of Σ9<110>38.94°, Σ5<110>109.47°, and Σ11<110>129.52° STGBs at the strain of (**a**,**d**,**g**) *ε* = 0; (**b**,**e**,**h**) *ε* = 0.01. The inset shows the angle between the upper grain and the GB plane, with the dashed line representing GB plane. (**c**), (**f**), and (**i**) correspond to the normal stress distribution of (**b**), (**e**) and (**h**), respectively. The grey and red balls represent Si and C atoms, respectively.

For <110>STGBs, three typical STGBs, Σ9<110>38.94°, Σ5<110>109.47°, and Σ11<100>129.52°, are selected. As illustrated in Figure 8, at the strain of *ε* = 0.01, the increments of the angle between the upper grain and the GB plane are 1.07°, 4.21°, and 1.36°, respectively, showing a trend of first increasing and then decreasing. The uniformity and magnitude variation in normal stress also indicate that as the misorientation angle approaches 90°, the shear strength decreases. In a previous study, Pang et al. [30] reported the theoretical shear strengths of five <110>STGBs in Al, calculated using ab initio methods. A similar result was observed along similar shear directions, i.e., the shear strength is symmetric with respect to the misorientation angle of 90°, which is thought to be related to the energy barrier for GB sliding. For <111> STGBs, no clear trend is observed in the relationship between shear strength and misorientation angle, reflecting the complex bonding characteristics at the interface.

### 3.4. Irradiation Response of GBs

#### 3.4.1. Evolution of Irradiation Defects

The irradiation response of bicrystals with various GB configurations was examined. During the initial phase of irradiation, known as the ballistic phase, several disordered regions with a large number of point defects are observed. After the number of defects reaches a peak, the recombination of interstitials and vacancies leads to a reduction in the number of defects in what is known as the thermally enhanced recovery phase. Finally, the number of defects stabilizes and fluctuates around a stable value.

**Figure 9:**
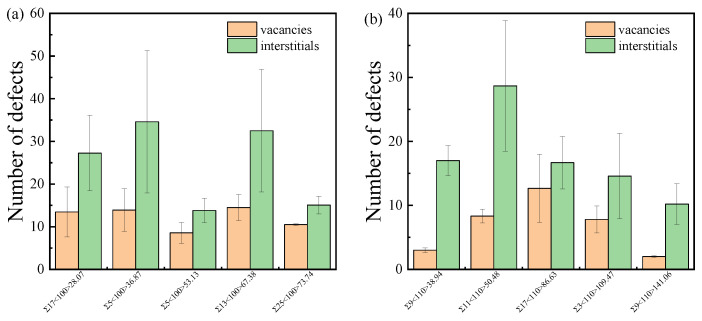
The number of survival vacancies and interstitials in the GB region for typical (**a**) <100> STGBs and (**b**) <110>STGBs. The standard deviations are shown with error bars.

Figure 9 illustrates the numbers of survival vacancies and interstitials in the GB region of ten typical low-Σ STGBs, where the GB region is determined by the potential energy described in Section 2.4. The error bars represent the standard deviations obtained from 10 separate simulations for each GB. The number of interstitials in the GB is greater than that of vacancies, which has been observed in bicrystals of other materials [65,66,67]. The GB can act as an efficient sink for interstitials, which is mainly attributed to (1) the diffusion coefficients towards GB and (2) the segregation energy of defects. According to the thermal activation mechanism, the diffusion coefficient of defect can be described by the Arrhenius relation *D* = *D*_0_exp(−*E*_m_*/k*_B_*T*), where *D*_0_ is the prefactor, *E*_m_ is the migration energy barrier, *k*_B_ is the Boltzmann constant, and *T* is the temperature. Ab initio calculations indicate that the migration barrier for C and Si vacancies are 3.66 eV and 2.70 eV, respectively. In contrast, the migration barrier for C and Si interstitials are 0.67 eV and 0.83 eV, respectively [68]. As a result, on the timescale of molecular dynamics simulations, the migration of vacancies at the temperature of 300 K is rare, while interstitials can diffuse into the GB region. The second reason is that vacancies and interstitials have different segregation energies to GBs. The segregation energy is defined as the difference in the formation energy of defects in the bulk compared to the GB. It is utilized to characterize the capture strength of GBs for point defects. Although the GB can absorb both vacancies and interstitials, interstitials exhibit a higher segregation energy and a larger segregation radius. Therefore, GB has a bias absorption effect on interstitials compared to vacancies. The phenomenon of carbon enrichment near GBs in irradiated SiC has been experimentally demonstrated [44]. The bias of interstitials in GBs leaves rich vacancies near the GB, which increases the rate of local amorphization, the so-called interstitial starvation mechanism [69].

Table 1 lists the structural characteristics and the number of survival defects in the GB region of ten typical low-Σ STGBs. The difference in the number of vacancies and interstitials (*δ*) has been utilized to characterize the efficiency of GB absorption as a measure of the resistance to irradiation of the system [67]. Findings from metallic bicrystal systems suggested that *δ* may be associated with the excess volume [70] or misorientation angle [67] of GBs. This study found no direct relationship between *δ* values and any GB characteristics. The sink efficiency of GBs for interstitials and vacancies is influenced by multiple factors. In SiC and other materials, the capability of GBs to trap interstitials is possibly related to the excess volume, interfacial energy, misorientation angle, etc. A larger excess volume corresponds to a more open GB structure, allowing it to accommodate atoms more easily [34,41]. The influence of GB energy [37,38] and misorientation angle [37] primarily arises from the dislocation structure of metallic GBs and the resulting changes in the GB stress field. On the other hand, the capacity of GBs to absorb vacancies may depend on their Σ values and energies; however, the impact of vacancies is significantly less than that of interstitials. SiC contains both Si and C atoms, and in the thermally enhanced recovery phase, the recovery of interstitials and vacancies is accompanied by the generation of antisite defects, which makes the evolution mechanism more complex.

**Figure 10:**
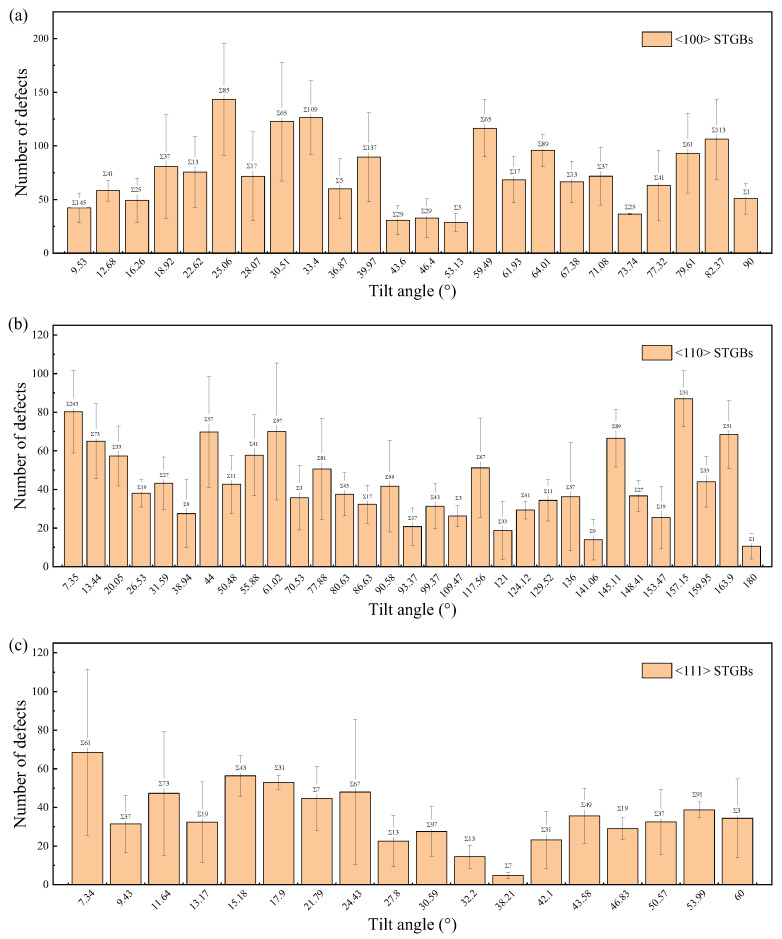
The number of defects in the GB region of STGBs as a function of misorientation angle with (**a**) <100> STGBs, (**b**) <110>STGBs, (**c**) <111> STGBs. The standard deviations are shown with error bars.

**Figure 11:**
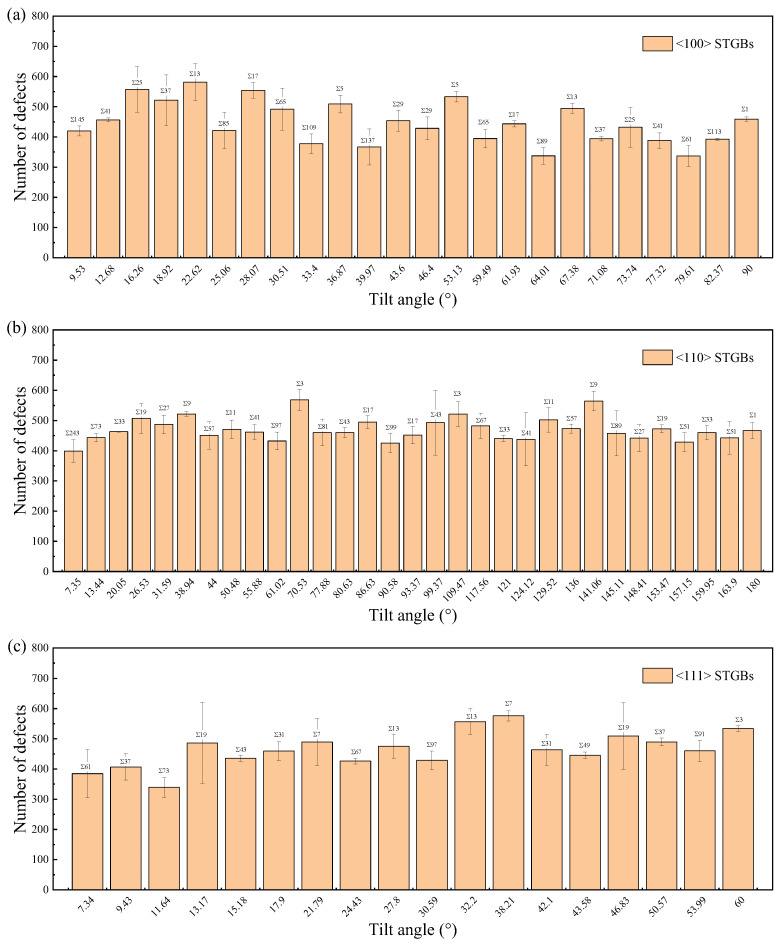
The total number of defects of STGBs as a function of misorientation angle with (**a**) <100> STGBs, (**b**) <110>STGBs, (**c**) <111> STGBs. The standard deviations are shown with error bars.

**Figure 12:**
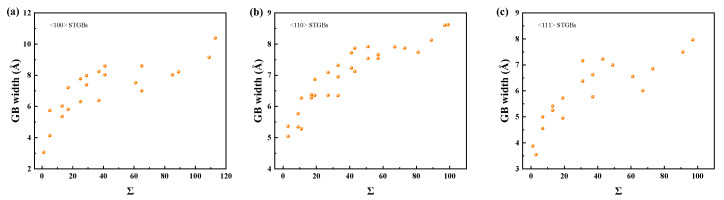
The GB width for STGBs as a function of Σ with (**a**) <100> STGBs, (**b**) <110>STGBs, (**c**) <111> STGBs.

The total number of survival defects, which is the sum of vacancies, interstitials, and antisite defects, is used to quantify the primary radiation damage. Figure 10 and Figure 11 illustrate the defect production in the GB regions and the substrates, respectively. It is found that the bicrystals with smaller Σ values survived a higher total number of defects, while fewer defects survived in the GB region. The number of defects in the GBs is related to their width, which generally increases with increasing Σ value, as shown in Figure 12. A GB with a lower Σ value tends to have a narrower GB width and consequently fewer defects in the GB region. Note that the assumption that the GB width does not change is taken when counting the number of defects [65,71]. Although the GB width expands after irradiation, this effect is negligible at low irradiation doses.

For GBs with smaller values of Σ, the number of defects in the grains is higher, which implies that more irradiation energy is dissipated in the grains rather than in the GBs. Since the atomic displacement threshold energy in GBs is lower than that in the bulk, it can be inferred that the total number of defects tends to be lower in bicrystals with smaller Σ values, which does not align with the results in Figure 11. It is observed that the distribution of defects in bicrystals with smaller Σ values is more discrete (Figure S4), which hinders the self-annihilation of defects. Additionally, the GB-induced cascade splitting probability [70] and the atomic displacement threshold energy in the GBs will also be different in different GBs. Ultimately, the number of survival defects results from multiple competing factors. In previous studies involving three and two STGB models in Nb, Manna et al. suggested that GBs with larger misorientation angles act as better sinks for defects, leading to fewer residual defects [72,73]. An attempt was made to explore the effect of GB characteristics on the production of irradiation defects based on nine SiC <100> STGB models (five of which were LAGBs) [64], but no general trend was found. As illustrated in Figure 10 and Figure 11, the variation in defect production corresponding to the Σ value is satisfied within a local angle range, which may not be captured when the number of GB types is limited.

The GB acts as a hard wall for a PKA to penetrate, thus reducing the maximum travel distance. For example, the average maximum travel distances of PKA in nanocrystalline and single crystalline SiC are 9.9 nm and 17.4 nm, respectively [74]. Most previous studies on bicrystals have focused on the overlap between the peak defect zone and the GB region. In this study, we are also concerned about the blocking effect of GBs on the transport of recoiled atoms, and therefore, a PKA of 8 keV Si is chosen to start the irradiation process so that the peak defect zone passes through the GB. The penetration distance *d*, defined as the distance from the farthest point defect generated in the upper grain to the initial position of the PKA, is calculated by the relationship *d* = (*x*^2^ + *y*^2^ + *z*^2^)^1/2^, where *x*, *y*, and *z* are the coordinates of the farthest point defect relative to the initial position of the PKA, respectively.

**Figure 13:**
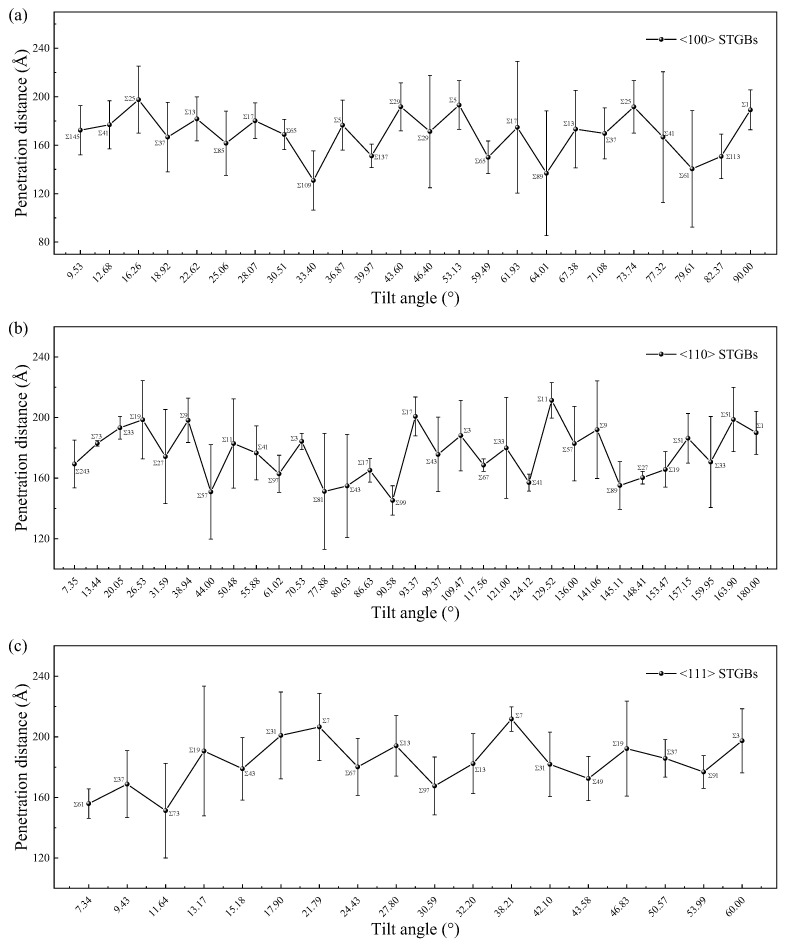
The penetration distance of STGBs as a function of misorientation angle with (**a**) <100> STGBs, (**b**) <110>STGBs, (**c**) <111> STGBs. The standard deviations are shown with error bars.

From Figure 13, it can be seen that the penetration distance of irradiation defects in bicrystals with smaller Σ values is relatively large, indicating a weaker GB blocking effect. Due to its thin GB region (as shown in Figure 12), cascades can penetrate the GB more easily and create more defects within the grain. Figure S4 demonstrates the blocking effect of GBs on irradiation defect propagation for different Σ values. A study of four STGBs in Fe also found that the Σ3 GB has the weakest ability to prevent cascade penetration [47]. Although the penetration distance is also influenced by the initial PKA direction, i.e., the orientation of the grain, it shows a clear dependence on the interfacial thickness rather than the misorientation angle. This means that for recoiled atoms with higher energies, the blocking ability of GB is primarily determined by its thickness. The blocking effects of different GBs on the cascade can reflect the localization degree of irradiation damage in polycrystalline materials, as well as the transition between intra-grain damage and GB damage, which can help the design of irradiation-resistant materials.

#### 3.4.2. Mechanical Response of Irradiated GBs

**Figure 14:**
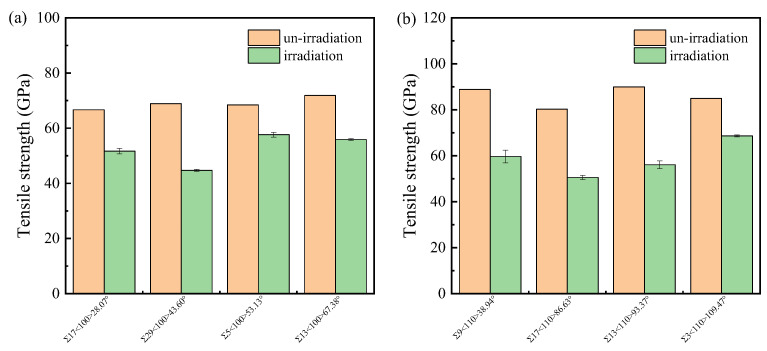
The tensile strength for typical (**a**) <100> STGBs and (**b**) <110>STGBs. The standard deviations are shown with error bars.

Once the irradiated system reaches equilibration, a uniaxial tensile strain is applied in the direction perpendicular to the GB plane to analyze the effect of irradiation damage on the mechanical properties under tensile loading. Figure S5a shows the typical stress–strain curves of both un-irradiated and irradiated bicrystal systems. The irradiated system still exhibits the brittle fracture behavior characteristic of ceramic materials. The tensile strength and related strain of the irradiated system decreased significantly compared to the pristine system. Statistical analysis of the tensile strength is illustrated in Figure 14. A recent study of nanocrystalline SiC reported that the tensile strength at different damage doses ranged from 21.1 to 41.6 GPa [54]. The difference is due to both the higher Σ values of some GBs in the nanocrystalline system and the difference in irradiation damage dose.

Figure S5b illustrates the structure of the bicrystalline system when fracture occurs. In the system with a small Σ value, crack nucleation preferentially occurs in the GB region rather than at the displacement cascade center in the upper grain, although the vacancy-rich zone at the cascade center also has weak resistance to external stresses. Regarding the effect of irradiation defects on the mechanical response of GBs in metallic materials, vacancy clusters promote the formation of microcracks, and interstitial clusters accelerate the nucleation of dislocation loos [47,66]. In SiC, the accumulation of defects at the GBs results in pronounced lattice disorder. As shown in Figure S6, C-C homonuclear bonds are formed in the GB region (*r* = 1.41 Å), even under single cascade collision. The increased level of chemical disorder will reduce the stability of the GB [54], leading to cracks sprouting in the GB.

**Figure 15:**
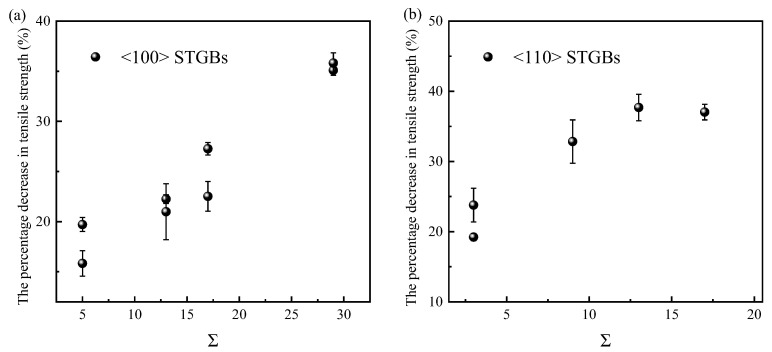
The percentage decrease in tensile strength after irradiation as a function of Σ values: (**a**) <100> STGBs, (**b**) <110>STGBs. The standard deviations are shown with error bars.

To quantitatively evaluate the effect of radiation damage on the reduction in tensile strength in different GB structures, some GBs with relatively small Σ values and small displacements during structure optimization are selected to calculate the average tensile strength after irradiation. The absolute tensile strength of the irradiated system is found not to be directly correlated with the GB structure, but the percentage decrease in tensile strength is related to the Σ value. This percentage decrease is calculated using the equation *η =* (*σ*_0_ − *σ*)/*σ*_0_, where *σ*_0_ and *σ* are the tensile strengths before and after irradiation, respectively. As shown in Figure 15, the percentage decrease in tensile strength positively correlates with the Σ value of the GB. According to Figure 10 and Figure 15, a smaller Σ value corresponds to a smaller number of survival defects in the GB region after irradiation, leading to a smaller percentage decrease in tensile strength. In previous studies of eight STGBs in Fe [47], it was found that the loss in tensile strength is high for Σ3 GBs and negligible for LAGBs, HAGBs, and Σ11GBs. The weakening of Σ3 GBs is thought to be associated with the rotation of interstitials towards the loading direction. In SiC, the tensile strength is closely related to the disorder of the GBs, so the number of defects has a significant effect. It should be pointed out that the aforementioned rule applies only to bicrystals with relatively low Σ values. In bicrystals with high Σ values, which are not shown in Figure 15, the concentrated distribution of defects in the upper grain can lead to cracks initiating in the vacancy-rich zone at the cascade center, rather than in the GB region.

For the shear response, radiation damage decreases the peak strength and the strain corresponding to the peak strength. However, no direct correlation is found between the reduction in shear strength and GB characteristics. One possible explanation is that defects at the GBs cause more pronounced bond fractures in the direction perpendicular to the GB plane. As a result, the influence of irradiation on the tensile response is more direct.

## 4. Conclusions

In this study, the energetics, mechanical properties, and irradiation response of STGBs with three tilt axes were investigated by MD simulations. The effect of GB characteristics (Σ value, misorientation angle, STGB system, etc.) on the STGB properties was focused on. Analyses show that GBs with small Σ values have lower energies and higher tensile strengths. The coherently confined Σ3<110>70.53° STGB exhibits the lowest GB energy, consistent with the experimental observation that the largest fractions of CSL GBs are the Σ3 GBs. The linear relationship between GB energy and excess volume in SiC is not as good as in metals or alloys because covalent bonding is more sensitive to orientation than metallic bonding. The shear strength is related to the positive stress near GB. For <100> STGBs, the shear strength is approximately linearly related to the misorientation angle. For <110>STGBs, the shear strength is approximately symmetric with respect to the misorientation angle of 90°, as previously reported in FCC metal computed with ab initio methods. The above variation pattern of shear strength is related to the uniformity and magnitude of the normal stress in the GB region. Some properties of <111> STGBs are different from those of <100> STGBs and <110>STGBs due to the complex bonding characteristics at the interface.

The GB has a bias absorption effect on interstitials compared to vacancies, but no direct correlation is found between the sink efficiency of GB for defects and GB characteristics. The STGBs with smaller Σ values survived a higher total number of defects, while fewer defects survived in the GB region, implying that more irradiation energy is dissipated in the grains rather than in the GBs. Note that the variation in defect production corresponding to the Σ value is satisfied within a local angle range, which may not be captured when the number of GB types is limited, and therefore, a systematic study is necessary. The mechanical response of irradiated STGBs shows that with the emergence of defects induced by irradiation, the homonuclear bonds can disrupt the chemical ordering of the original heteronuclear bonds, causing cracks to sprout at GBs and degrading the structural strength. Interestingly, the percentage decrease in tensile strength of the irradiated bicrystal is positively correlated with the Σ value. However, the relationship between the shear response and the GB type remains to be further studied.

Overall, the analyses in the present work have established the correlation between GB characteristics and their behaviors. It should be noted that these results were obtained at a temperature of 300 K. Further investigation is needed to determine if these trends still hold at higher temperatures in nuclear reactors. The deformation mechanism of a material is dependent not only on its characteristics but also on the temperature. Meanwhile, elevated temperatures influence material behavior during irradiation, leading to structural disorders and increased kinetic energies of recoiled atoms. These combined factors result in changes in penetration distance, the distribution of irradiation defects, etc. The effects of higher temperatures will be assessed in future simulations.

## Figures and Tables

**Table 1:** The misorientation angles, Σ values, GB energies, excess volumes, GB widths, average number of survival vacancies and interstitials, and difference in the number of vacancies and interstitials (δ) in the GB region of ten typical STGBs.

STGB System	*θ* (°)	Σ	GB Energy (J/m^2^)	Excess Volume (J/m^2^)	GB Width (Å)	*N*_v_	*N*_i_	*δ*
<100>	28.07	17	2.69	0.66	4.42	13	27	14
<100>	36.87	5	2.63	0.58	4.64	14	35	21
<100>	53.13	5	2.61	0.74	4.86	9	14	5
<100>	67.38	13	2.56	0.61	5.42	15	33	18
<100>	73.74	25	2.50	0.59	6.40	11	15	4
<110>	38.94	9	1.44	0.13	4.36	3	17	14
<110>	50.48	11	2.15	0.36	5.56	8	29	21
<110>	86.63	17	2.12	0.34	5.20	13	17	4
<110>	109.47	3	2.04	0.30	3.54	8	15	7
<110>	141.06	9	1.63	0.34	4.08	2	10	8

## Data Availability

The raw data supporting the conclusions of this article will be made available by the authors on request.

## Supplementary Materials

The following supporting information can be downloaded at: https://www.mdpi.com/article/10.3390/ma18153545/s1, Figure S1: The stress-strain curves of STGBs under uniaxial tensile loading at *T* = 300 K: (a) <100> STGBs, (b) <110>STGBs, and (c) <111> STGBs; Figure S2: The structure of Σ51<110>157.15° STGB: (a) before optimization, (b) after optimization. The blue, red and white balls represent the atoms in the upper grain, lower grain and GB plane, respectively; Figure S3: The stress-strain curves of STGBs under shear loading at *T* = 300 K: (a) <100> STGBs, (b) <110>STGBs, and (c) <111> STGBs; Figure S4: The distribution of defects in (a) Σ29<100>46.40° STGB and (b) Σ5<100>53.13° STGB at *E*_PKA_ = 8 keV and *T* = 300 K, with the dashed line representing GB plane; Figure S5: (a) The stress–strain curves of typical STGBs under uniaxial tensile loading at *T* = 300 K before and after *E*_PKA_ = 8 keV irradiation. (b) The structure at the time of fracture. The red and gray balls represent C and Si atoms, respectively; Figure S6: The radial distribution function curves before and after irradiation in the GB region of Σ29<100>43.60° STGB. The arrow points to the formation of C–C homonuclear bond; Table S1: The misorientation angle (*θ*), Σ value, the dimensions of the simulation box (*x*, *y*, *z*) and the number of atoms (*N*) of the <100> STGBs models in the simulation of GB energy; Table S2: The misorientation angle (*θ*), Σ value, the dimensions of the simulation box (*x*, *y*, *z*) and the number of atoms (*N*) of the <110>STGBs models in the simulation of GB energy; Table S3: The misorientation angle (*θ*), Σ value, the dimensions of the simulation box (*x*, *y*, *z*) and the number of atoms (*N*) of the <111> STGBs models in the simulation of GB energy; Table S4: The misorientation angle (*θ*), Σ value, the dimensions of the simulation box (*x*, *y*, *z*) and the number of atoms (*N*) of the <100> STGBs models in the simulation of mechanical properties; Table S5: The misorientation angle (*θ*), Σ value, the dimensions of the simulation box (*x*, *y*, *z*) and the number of atoms (*N*) of the <110>STGBs models in the simulation of mechanical properties; Table S6: The misorientation angle (*θ*), Σ value, the dimensions of the simulation box (*x*, *y*, *z*) and the number of atoms (*N*) of the <111> STGBs models in the simulation of mechanical properties; Table S7: The misorientation angle (*θ*), Σ value, the dimensions of the simulation box (*x*, *y*, *z*) and the number of atoms (*N*) of the <100> STGBs models in the simulation of irradiation; Table S8: The misorientation angle (*θ*), Σ value, the dimensions of the simulation box (*x*, *y*, *z*) and the number of atoms (*N*) of the <110>STGBs models in the simulation of irradiation; Table S9: The misorientation angle (*θ*), Σ value, the dimensions of the simulation box (*x*, *y*, *z*) and the number of atoms (*N*) of the <111> STGBs models in the simulation of irradiation.

## References

[1] Duchnowski E.M., Brown N.R. (2024). A review of multiphysics tools and methods to evaluate high temperature pebble bed reactors. Prog. Nucl. Energy.

[2] Brown N.R. (2020). A review of in-pile fuel safety tests of TRISO fuel forms and future testing opportunities in non-HTGR applications. J. Nucl. Mater..

[3] Katoh Y., Snead L.L. (2019). Silicon carbide and its composites for nuclear applications—Historical overview. J. Nucl. Mater..

[4] Chen Z., Huang A.Q. (2024). Extreme high efficiency enabled by silicon carbide (SiC) power devices. Mater. Sci. Semicond. Process..

[5] Wei J., Wei Z., Fu H., Cao J., Wu T., Sun J., Zhu X., Li S., Zhang L., Liu S. (2023). Review on the Reliability Mechanisms of SiC Power MOSFETs: A Comparison Between Planar-Gate and Trench-Gate Structures. IEEE Trans. Power Electron..

[6] Haque Chowdhury E., Habibur Rahman M., Hong S. (2021). Tensile strength and fracture mechanics of two-dimensional nanocrystalline silicon carbide. Comput. Mater. Sci..

[7] Pabst O., Schiffer M., Obermeier E., Tekin T., Lang K.D., Ngo H.D. (2012). Measurement of Young’s modulus and residual stress of thin SiC layers for MEMS high temperature applications. Microsyst. Technol..

[8] Liao F., Girshick S.L., Mook W.M., Gerberich W.W., Zachariah M.R. (2005). Superhard nanocrystalline silicon carbide films. Appl. Phys. Lett..

[9] Kulikovsky V., Vorlíček V., Boháč P., Stranyánek M., Čtvrtlík R., Kurdyumov A., Jastrabik L. (2008). Hardness and elastic modulus of amorphous and nanocrystalline SiC and Si films. Surf. Coat. Technol..

[10] Espinoza-Pérez L.J., Esquivel-Medina S., López-Honorato E. (2021). Influence of SiC microstructure on its corrosion behavior in molten FLiNaK salt. Ceram. Int..

[11] Liu C., Xi J., Szlufarska I. (2019). Sensitivity of SiC Grain Boundaries to Oxidation. J. Phys. Chem. C.

[12] Cancino-Trejo F., López-Honorato E., Walker R.C., Ferrer R.S. (2018). Grain-boundary type and distribution in silicon carbide coatings and wafers. J. Nucl. Mater..

[13] Sinha S., Kim D.-I., Fleury E., Suwas S. (2015). Effect of grain boundary engineering on the microstructure and mechanical properties of copper containing austenitic stainless steel. Mater. Sci. Eng. A.

[14] Safari H., Rezaeian A., Karimzadeh F. (2024). Novel role of thermomechanical grain boundary engineering in the microstructure evolution of austenitic stainless steel. J. Mater. Res. Technol..

[15] Xi X., Liu Z., Qin Z., Wu T., Wang J., Xu N., Chen L. (2023). Enhancement of the resistance to hydrogen embrittlement by tailoring grain boundary characteristics in a low carbon high strength steel. J. Mater. Res. Technol..

[16] Kohler C. (2002). Atomistic Modelling of Structures of Tilt Grain Boundaries and Antiphase Boundaries in β-Silicon Carbide. Phys. Status Solidi B.

[17] Wojdyr M., Khalil S., Liu Y., Szlufarska I. (2010). Energetics and structure of ⟨0 0 1⟩ tilt grain boundaries in SiC. Model. Simul. Mater. Sci..

[18] Jin E., Niu L.S., Lin E., Song X. (2012). Grain boundary effects on defect production and mechanical properties of irradiated nanocrystalline SiC. J. Appl. Phys..

[19] Bringuier S., Manga V.R., Runge K., Deymier P., Muralidharan K. (2015). Grain boundary dynamics of SiC bicrystals under shear deformation. Mater. Sci. Eng. A.

[20] Li Y., Li Y., Xiao W. (2019). Point defects and grain boundary effects on tensile strength of 3C-SiC studied by molecular dynamics simulations. Nucl. Eng. Technol..

[21] Guziewski M., Banadaki A.D., Patala S., Coleman S.P. (2020). Application of Monte Carlo techniques to grain boundary structure optimization in silicon and silicon-carbide. Comput. Mater. Sci..

[22] Wang L., Zhang L., Yu W. (2024). Revisiting the structures and energies of β-SiC <001> symmetric tilt grain boundaries. J. Mater. Res..

[23] Montes de Oca Zapiain D., Guziewski M., Coleman S.P., Dingreville R. (2020). Characterizing the Tensile Strength of Metastable Grain Boundaries in Silicon Carbide Using Machine Learning. J. Phys. Chem. C.

[24] Guziewski M., Montes de Oca Zapiain D., Dingreville R., Coleman S.P. (2021). Microscopic and Macroscopic Characterization of Grain Boundary Energy and Strength in Silicon Carbide via Machine-Learning Techniques. ACS Appl. Mater. Interfaces.

[25] Gur S., Sadat M.R., Frantziskonis G.N., Bringuier S., Zhang L., Muralidharan K. (2019). The effect of grain-size on fracture of polycrystalline silicon carbide: A multiscale analysis using a molecular dynamics-peridynamics framework. Comput. Mater. Sci..

[26] Han Y.S., Tomar V. (2015). An investigation into the influence of grain boundary misorientation on the tensile strength of SiC bicrystals. Mech. Adv. Mater. Struct..

[27] Song H.Y., Li C.F., Geng S.F., An M.R., Xiao M.X., Wang L. (2018). Atomistic simulations of effect of hydrogen atoms on mechanical behaviour of an α -Fe with symmetric tilt grain boundaries. Phys. Lett. A.

[28] Wang K., Xu Y., Zhang W., Xu J. (2023). The impact of structural units on the dislocation nucleation of bi-crystal copper grain boundary. Comput. Mater. Sci..

[29] Bomarito G.F., Lin Y., Warner D.H. (2015). An atomistic modeling survey of the shear strength of twist grain boundaries in aluminum. Scr. Mater..

[30] Pang X., Ahmed N., Janisch R., Hartmaier A. (2012). The mechanical shear behavior of Al single crystals and grain boundaries. J. Appl. Phys..

[31] Shao M., Xu C., Hu R., Lang Z., Li P., Wang Z., Liu H., Liu C. (2025). Damping effect of (110)<001> symmetric tilt grain boundaries on the shock response of SiC. Surf. Interfaces.

[32] Field K.G., Yang Y., Allen T.R., Busby J.T. (2015). Defect sink characteristics of specific grain boundary types in 304 stainless steels under high dose neutron environments. Acta Mater..

[33] Nathaniel J.E., Suri P.K., Hopkins E.M., Wen J., Baldo P., Kirk M., Taheri M.L. (2022). Grain boundary strain as a determinant of localized sink efficiency. Acta Mater..

[34] Sun J., You Y.-W., Wu X., Song H.-Y., Li B.S., Liu C.S., Krsjak V. (2022). Segregation and diffusion behaviours of helium at grain boundaries in silicon carbide ceramics: First-principles calculations and experimental investigations. J. Eur. Ceram. Soc..

[35] Jiang H., Wang X., Szlufarska I. (2017). The Multiple Roles of Small-Angle Tilt Grain Boundaries in Annihilating Radiation Damage in SiC. Sci. Rep..

[36] Meng Z., Wang C., Wang Y., Liu Y., Shu Y., Yang L. (2023). Screening and manipulation by segregation of dopants in grain boundary of Silicon carbide: First-principles calculations. Ceram. Int..

[37] Li X., Wang Y., Zhang Y., Xu Y., Li X.-Y., Wang X., Fang Q.F., Wu X., Liu C.S. (2022). Towards the dependence of radiation damage on the grain boundary character and grain size in tungsten: A combined study of molecular statics and rate theory. J. Nucl. Mater..

[38] Xu C., Tian X., Jiang W., Wang Q., Fan H. (2024). The sink efficiency of symmetric tilt grain boundary under displacement cascade in zirconium. J. Nucl. Mater..

[39] Jiang H., Szlufarska I. (2018). Small-Angle Twist Grain Boundaries as Sinks for Point Defects. Sci. Rep..

[40] Tschopp M.A., Solanki K.N., Gao F., Sun X., Khaleel M.A., Horstemeyer M.F. (2012). Probing grain boundary sink strength at the nanoscale: Energetics and length scales of vacancy and interstitial absorption by grain boundaries in α-Fe. Phys. Rev. B.

[41] He W.-H., Gao X., Gao N., Wang J., Wang D., Cui M.-H., Pang L.-L., Wang Z.-G. (2018). Effects of Grain Boundary Characteristics on Its Capability to Trap Point Defects in Tungsten. Chin. Phys. Lett..

[42] Wang Z., Zhang L., AlMotasem A.T., Li B., Polcar T., Daghbouj N. (2024). Exploring defect behavior in helium-irradiated single-crystal and nanocrystalline 3C-SiC at 800 °C: A synergy of experimental and simulation techniques. Acta Mater..

[43] Cui Y., Sun J., Li M., Li B. (2025). First-Principles Calculations of Hydrogen Solution and Diffusion in 3C-SiC Grain Boundaries. Materials.

[44] Wang X., Zhang H., Baba T., Jiang H., Liu C., Guan Y., Elleuch O., Kuech T., Morgan D., Idrobo J.C. (2020). Radiation-induced segregation in a ceramic. Nat. Mater..

[45] Han Y.S., Tomar V. (2014). An ab-initio analysis of the influence of knock-on atom induced damage on the peak tensile strength of 3C-SiC grain boundaries. Int. J. Damage Mech..

[46] Han Y.S., Tomar V. (2014). An ab initio study of the structure–strength correlation in impact damaged SiC grain boundaries. Comput. Mater. Sci..

[47] Kedharnath A., Kapoor R., Sarkar A. (2019). Atomistic simulation of interaction of collision cascade with different types of grain boundaries in α-Fe. J. Nucl. Mater..

[48] Torres E., Maxwell C. (2023). Effect of irradiation damage on the tensile deformation of α-zirconium systems: A molecular dynamics study. Comput. Mater. Sci..

[49] Plimpton S. (1995). Fast Parallel Algorithms for Short-Range Molecular Dynamics. J. Comput. Phys..

[50] Stukowski A. (2010). Visualization and analysis of atomistic simulation data with OVITO–the Open Visualization Tool. Model. Simul. Mater. Sci. Eng..

[51] Tersoff J. (1989). Modeling solid-state chemistry: Interatomic potentials for multicomponent systems. Phys. Rev. B.

[52] Ziegler J.F., Biersack J.P., Bromley D.A. (1985). The Stopping and Range of Ions in Matter. Treatise on Heavy-Ion Science.

[53] Devanathan R., Weber W.J., De La Rubia T.D. (1998). Computer simulation of a 10 keV Si displacement cascade in SiC. Nucl. Instrum. Methods Phys. Res. Sect. B Beam Interact. Mater. At..

[54] Cai Z., Yuan X., Xu C., Li Y., Shao Z., Li W., Xu J., Zhang Q. (2024). Grain boundary effects on chemical disorders and amorphization-induced swelling in 3C-SiC under high-temperature irradiation: From atomic simulation insight. J. Eur. Ceram. Soc..

[55] Gao F., Weber W.J. (2002). Cascade overlap and amorphization in3C−SiC:Defect accumulation, topological features, and disordering. Phys. Rev. B.

[56] Swaminathan N., Kamenski P.J., Morgan D., Szlufarska I. (2010). Effects of grain size and grain boundaries on defect production in nanocrystalline 3C–SiC. Acta Mater..

[57] Tong X., Zhang H., Li D. (2014). Effects of misorientation and inclination on mechanical response of 〈1 1 0〉 tilt grain boundaries inα-Fe to external stresses. Model. Simul. Mater. Sci. Eng..

[58] Hallil A., Metsue A., Bouhattate J., Feaugas X. (2016). Correlation between vacancy formation and Σ3 grain boundary structures in nickel from atomistic simulations. Philos. Mag..

[59] Uesugi T., Higashi K. (2011). First-principles calculation of grain boundary energy and grain boundary excess free volume in aluminum: Role of grain boundary elastic energy. J. Mater. Sci..

[60] Pal S., Reddy K.V., Yu T., Xiao J., Deng C. (2021). The spectrum of atomic excess free volume in grain boundaries. J. Mater. Sci..

[61] Xing Z., Fan H., Xu C., Kang G. (2024). Transition from grain boundary migration to grain boundary sliding in magnesium bicrystals. Acta Mech. Sin..

[62] Hua A., Zhao J. (2022). Shear direction induced transition mechanism from grain boundary migration to sliding in a cylindrical copper bicrystal. Int. J. Plast..

[63] Yang L., Song X., Yu T., Liu D., Deng C. (2024). Unusual acceleration and size effects in grain boundary migration with shear coupling. Comput. Mater. Sci..

[64] Swaminathan N., Wojdyr M., Morgan D.D., Szlufarska I. (2012). Radiation interaction with tilt grain boundaries in β-SiC. J. Appl. Phys..

[65] Wang X.Y., Gao N., Setyawan W., Xu B., Liu W., Wang Z.G. (2017). Effect of irradiation on mechanical properties of symmetrical grain boundaries investigated by atomic simulations. J. Nucl. Mater..

[66] Lin P., Nie J., Lu Y., Xiao G., Gu G., Cui W., He L. (2024). Effect of irradiation on mechanical properties of symmetrical grain boundaries in BCC tungsten: An atomistic study. Appl. Phys. A.

[67] Li B., Long X.-J., Shen Z.-W., Luo S.-N. (2016). Interactions between displacement cascades and Σ3<110> tilt grain boundaries in Cu. J. Nucl. Mater..

[68] Shrader D., Khalil S.M., Gerczak T., Allen T.R., Heim A.J., Szlufarska I., Morgan D. (2011). Ag diffusion in cubic silicon carbide. J. Nucl. Mater..

[69] Wang X., Jamison L., Sridharan K., Morgan D., Voyles P.M., Szlufarska I. (2015). Evidence for cascade overlap and grain boundary enhanced amorphization in silicon carbide irradiated with Kr ions. Acta Mater..

[70] Wang H., Zhou Y., Dai L., Mi X., Sun C., Dong Q., Wu L., Tan J., Tang A. (2023). Interaction of displacement cascades with {10 1^−^2} and {10 1^−^1} twin boundaries in zirconium: A molecular dynamic study. J. Mater. Res. Technol..

[71] Li H., Qin Y., Yang Y., Yao M., Wang X., Xu H., Phillpot S.R. (2018). The evolution of interaction between grain boundary and irradiation-induced point defects: Symmetric tilt GB in tungsten. J. Nucl. Mater..

[72] Manna M., Pal S. (2023). Molecular dynamics simulation for radiation response of Nb bicrystal having Σ 13, Σ 29, and Σ 85 grain boundary. J. Appl. Phys..

[73] Manna M., Pal S. (2025). Irradiation Damage Evolution Dependence on Misorientation Angle for Σ 5 Grain Boundary of Nb: An Atomistic Simulation-Based Study. J. Eng. Mater. Technol..

[74] Gao F., Chen D., Hu W., Weber W.J. (2010). Energy dissipation and defect generation in nanocrystalline silicon carbide. Phys. Rev. A.

