# Supplementary material for: Effect of Grain Boundary Characteristics on Mechanical Properties and Irradiation Response in 3C-SiC: A Molecular Dynamics Simulation Study
Source: Materials (Basel). 2025 Jul 29;18(15):3545. doi: 10.3390/ma18153545 (PMC12348254; doi:10.3390/ma18153545)
Supplement: Supplementary file 1 [file materials-18-03545-s001.zip › materials-3718811-supplementary.pdf]

Supplementary material

Effect of grain boundary characteristics on mechanical properties and irradiation response in 3C-SiC: A molecular dynamics simulation study

Wenying Liu [a], Fugen Deng[1], Jiajie Yu[1], Lin Chen [a], Yuyang Zhou [a], Yulu Zhou [a, *], Yifang Ouyang [a]

[a] *School of Physical Science and Technology, State Key Laboratory of Featured Metal Materials and Life-cycle Safety for Composite Structures, Guangxi Key Laboratory for Relativistic Astrophysics, Guangxi University, Nanning 530004, China*

* Correspondence: ylzhou@gxu.edu.cn

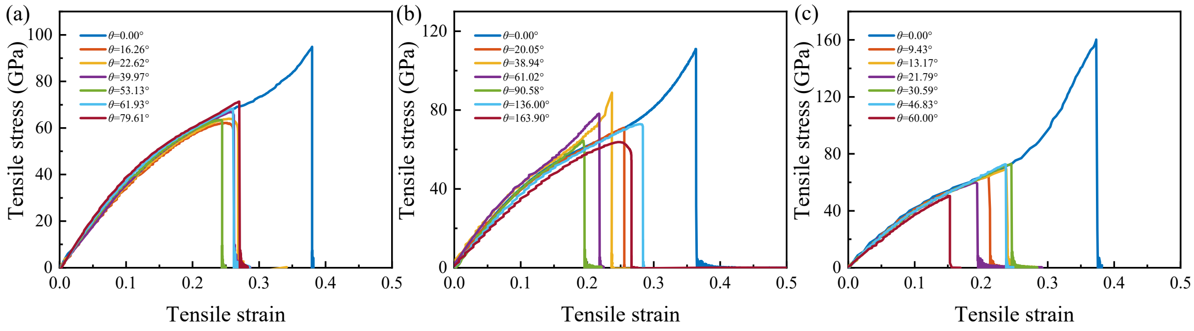

Figure S1. The stress-strain curves of STGBs under uniaxial tensile loading at $T = 300$ K: (a) <100> STGBs, (b) <110> STGBs, and (c) <111> STGBs.

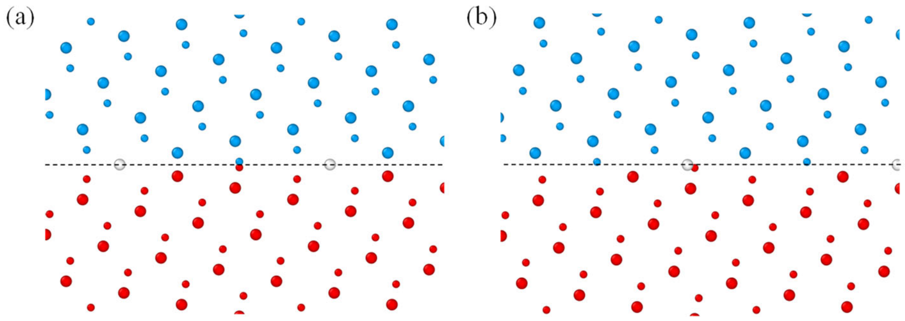

Figure S2. The structure of Σ51<110>157.15° STGB: (a) before optimization, (b) after optimization. The blue, red and white balls represent the atoms in the upper grain, lower grain and GB plane, respectively.

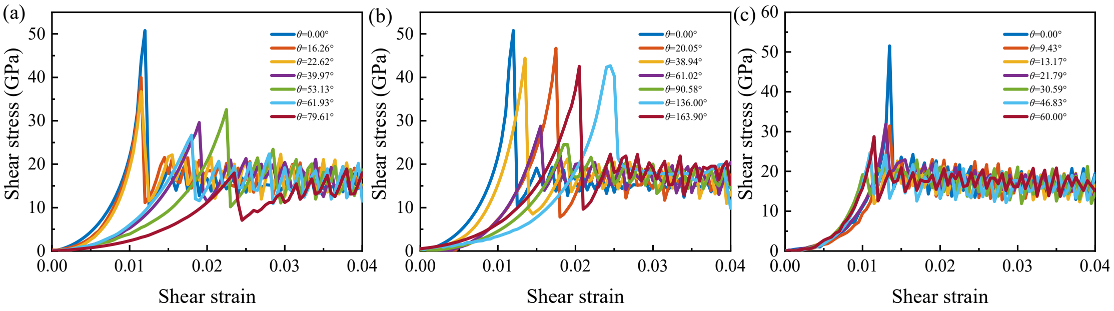

Figure S3. The stress-strain curves of STGBs under shear loading at $T = 300$ K: (a) <100> STGBs, (b) <110> STGBs, and (c) <111> STGBs.

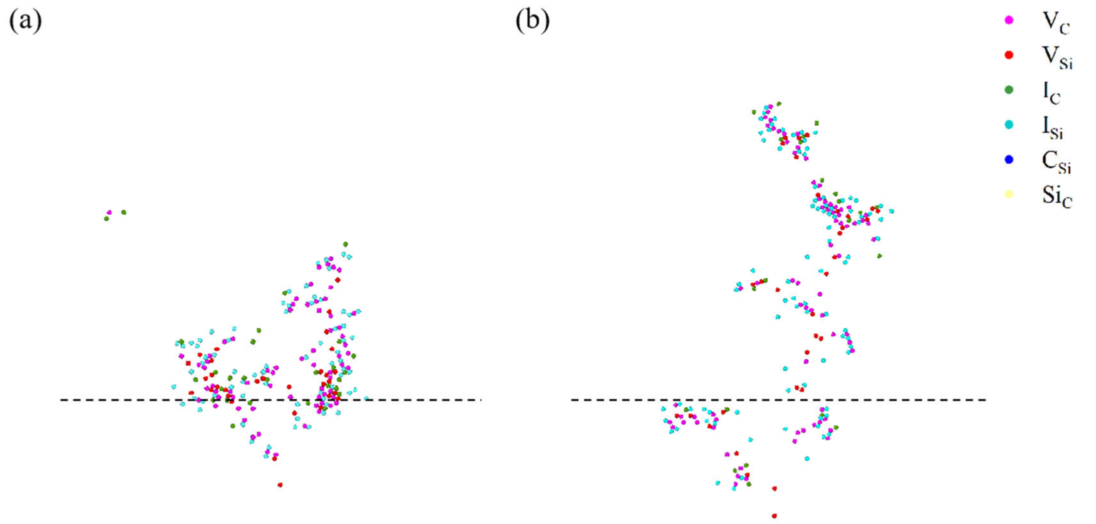

Figure S4. The distribution of defects in (a) Σ29<100>46.40° STGB and (b) Σ5<100>53.13° STGB at E_{PKA} = 8 keV and T = 300 K, with the dashed line representing GB plane.

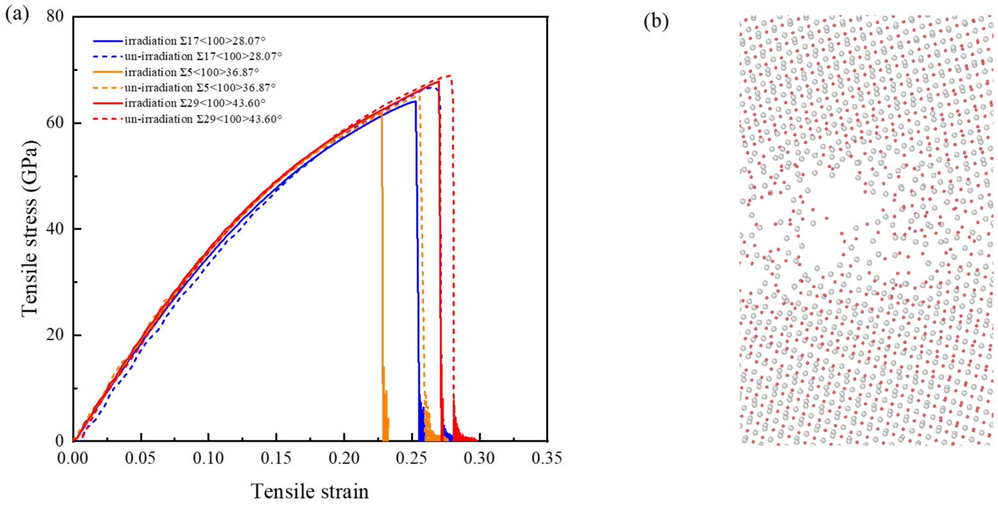

Figure S5. (a) The stress–strain curves of typical STGBs under uniaxial tensile loading at T = 300 K before and after E_{PKA} = 8 keV irradiation. (b) The structure at the time of fracture. The red and gray balls represent C and Si atoms, respectively.

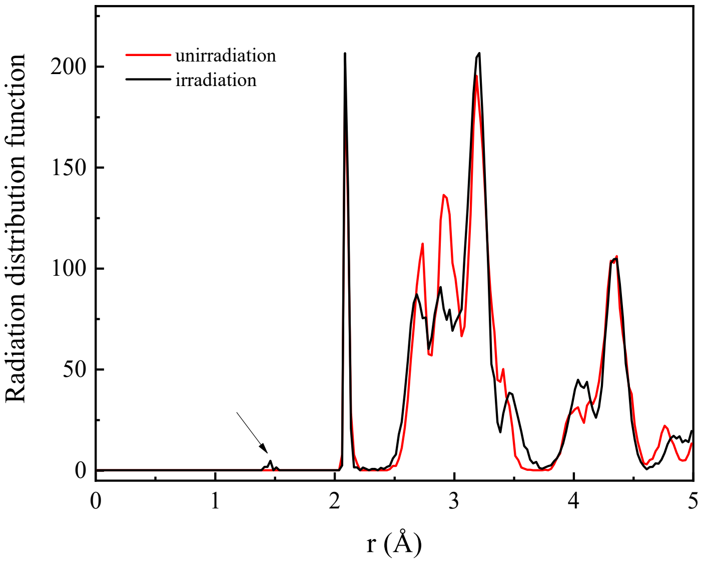

Figure S6. The radial distribution function curves before and after irradiation in the GB region of Σ29<100>43.60° STGB. The arrow points to the formation of C–C homonuclear bond.

Table S1. The misorientation angle (θ), Σ value, the dimensions of the simulation box (x, y, z) and the number of atoms (N) of the <100> STGBs models in the simulation of GB energy.

θ (°)	Σ	x (Å)	y (Å)	z (Å)	N
0.00	1	52.17	104.34	21.74	11640
9.53	145	52.36	104.72	21.74	11600
12.68	41	59.05	118.10	21.74	14780
16.26	25	61.49	122.98	21.74	16030
18.92	37	52.89	105.78	21.74	11860
22.62	13	55.42	110.84	21.74	13030
25.06	85	80.17	160.34	13.04	16332
28.07	17	53.78	107.56	21.74	12440
30.51	65	49.58	99.16	21.74	10410
33.40	109	45.39	90.78	26.09	10476
36.87	5	55.00	110.00	21.74	12830
39.97	137	50.89	101.78	21.74	10970
43.60	29	46.83	93.66	26.09	11160
46.40	29	49.67	99.34	21.74	10460
53.13	5	48.61	97.22	21.74	10050
59.49	65	70.11	140.22	21.74	20820
61.93	17	50.70	101.40	21.74	10910
64.01	89	82.03	164.06	21.74	28470
67.38	13	62.70	125.40	21.74	16680
71.08	37	56.10	112.20	21.74	13320
73.74	25	65.22	130.44	21.74	18030
77.32	41	55.68	111.36	21.74	13140
79.61	61	67.91	135.82	21.74	19520
82.37	113	46.22	92.44	26.09	10860
90.00	1	49.19	98.38	21.74	10320

Table S2. The misorientation angle (θ), Σ value, the dimensions of the simulation box (x, y, z) and the number of atoms (N) of the <110> STGBs models in the simulation of GB energy.

θ (°)	Σ	x (Å)	y (Å)	z (Å)	N
0.00	1	43.48	173.92	24.59	10400
7.35	243	47.93	135.56	24.59	15564
13.44	73	52.53	111.44	24.59	14022
20.05	33	52.98	99.90	24.59	12687
26.53	19	40.20	113.72	24.59	10950
31.59	27	45.18	95.84	24.59	10389
38.94	9	46.11	91.30	24.59	10112
44.00	57	65.65	92.84	24.59	14609
50.48	11	43.26	101.96	24.59	10602
55.88	41	59.06	111.36	24.59	15764
61.02	97	60.56	128.46	18.44	13980
70.53	3	47.93	105.44	24.59	12070
77.88	81	78.26	110.68	24.59	20763
80.63	43	57.02	120.96	18.44	12385
86.63	17	53.78	101.40	24.59	13080
90.58	99	86.52	122.36	24.59	25376
93.37	17	50.70	107.56	24.59	13068
99.37	43	40.32	114.04	24.59	11008
109.47	3	45.18	95.86	24.59	10355
117.56	67	50.33	106.76	24.59	12914
121.00	33	52.98	99.90	24.59	12691
124.12	41	55.68	118.12	18.44	11819
129.52	11	40.79	115.36	24.59	11279
136.00	57	46.42	98.48	24.59	10960
141.06	9	52.18	110.68	24.59	13850
145.11	89	58.01	123.06	18.44	12828
148.41	27	47.93	112.96	24.59	12954
153.47	19	56.85	107.20	24.59	14585
157.15	51	43.91	93.16	30.74	12284
159.95	33	49.95	105.96	24.59	12696
163.90	51	62.10	131.74	18.44	14700
180.00	1	55.34	121.74	18.45	12204

Table S3. The misorientation angle (θ), Σ value, the dimensions of the simulation box (x, y, z) and the number of atoms (N) of the <111> STGBs models in the simulation of GB energy.

θ (°)	Σ	x (Å)	y (Å)	z (Å)	N
0.00	1	42.60	110.68	22.59	10512
7.34	61	72.03	166.36	15.06	17572
9.43	37	56.10	129.56	22.59	15988
11.64	73	45.50	105.08	22.59	10523
13.17	19	40.20	139.26	22.59	12319
15.18	43	69.84	120.96	22.59	18600
17.90	31	59.30	136.94	22.59	17873
21.79	7	73.21	112.72	15.06	12114
24.43	67	43.59	100.66	30.12	12872
27.80	13	57.60	133.02	15.06	11252
30.59	97	52.59	182.18	30.20	27928
32.20	13	66.51	115.20	22.59	16872
38.21	7	56.36	97.60	22.59	12133
42.10	31	51.35	118.60	22.59	13403
43.57	49	74.55	129.12	22.59	21170
46.83	19	46.42	107.20	22.59	10959
50.57	37	64.78	149.60	22.59	21336
53.99	91	50.80	117.32	22.59	13108
60.00	3	55.34	85.20	22.59	10357

Table S4. The misorientation angle (θ), Σ value, the dimensions of the simulation box (x, y, z) and the number of atoms (N) of the <100> STGBs models in the simulation of mechanical properties.

θ (°)	Σ	x (Å)	y (Å)	z (Å)	N
0.00	1	43.48	95.66	43.48	35400
9.53	145	52.35	104.71	39.13	41760
12.68	41	59.06	78.74	39.13	35460
16.26	25	46.12	92.23	43.48	36040
18.92	37	52.89	105.79	43.48	47400
22.62	13	44.34	88.68	47.82	36674
25.06	85	40.09	80.17	56.52	35386
28.07	17	53.78	89.63	52.32	49032
30.51	65	49.57	99.15	39.13	37458
33.40	109	45.39	90.79	43.48	34900
36.87	5	54.99	96.25	39.13	40374
39.97	137	50.89	101.78	39.13	39474
43.60	29	46.83	93.66	47.83	40876
46.40	29	49.67	99.34	39.13	37620
53.13	5	48.61	87.50	47.83	39710
59.49	65	52.58	70.11	52.18	37476
61.93	17	50.70	101.41	47.83	47938
64.01	89	41.02	82.04	52.18	34176
67.38	13	47.03	94.06	47.83	41250
71.08	37	56.10	74.80	43.48	35520
73.74	25	43.48	86.96	47.83	35244
77.32	41	55.68	83.52	43.48	39400
79.61	61	67.92	101.87	39.13	52704
82.37	113	46.21	92.44	43.48	36180
90.00	1	49.19	86.07	43.48	36160

Table S5. The misorientation angle (θ), Σ value, the dimensions of the simulation box (x, y, z) and the number of atoms (N) of the <110> STGBs models in the simulation of mechanical properties.

θ (°)	Σ	x (Å)	y (Å)	z (Å)	N
0.00	1	43.48	92.23	49.19	38720
7.35	243	47.93	101.67	43.04	40819
13.44	73	52.54	74.30	49.19	37392
20.05	33	52.98	99.91	43.04	44375
26.53	19	53.61	94.76	43.04	42607
31.59	27	45.18	95.85	43.04	36324
38.94	9	46.12	91.31	43.04	35334
44.00	57	65.65	92.85	43.04	51105
50.48	11	57.68	81.58	49.19	45162
55.88	41	59.06	83.52	49.19	47275
61.02	97	60.56	85.65	49.19	49691
70.53	3	42.60	97.90	43.04	35046
77.88	81	78.26	83.01	43.04	54479
80.63	43	57.02	80.64	43.04	38534
86.63	17	53.78	88.74	43.04	40022
90.58	99	43.26	91.77	49.19	38047
93.37	17	50.70	89.63	49.19	43555
99.37	43	60.48	71.27	43.04	36013
109.47	3	45.19	85.20	49.19	36964
117.56	67	50.33	71.18	55.34	38635
121.00	33	52.98	74.93	49.19	38054
124.12	41	55.68	78.75	43.04	36768
129.52	11	50.98	86.52	43.04	37006
136.00	57	46.42	98.48	43.04	38332
141.06	9	52.18	92.23	43.04	40371
145.11	89	58.01	82.04	49.19	45595
148.41	27	47.92	90.37	43.04	36353
153.47	19	56.86	80.41	43.04	38365
157.15	51	43.91	93.15	49.19	39176
159.95	33	49.95	70.65	55.34	38071
163.90	51	62.10	87.83	43.04	45713
180.00	1	46.12	95.65	43.04	37170

Table S6. The misorientation angle (θ), Σ value, the dimensions of the simulation box (x, y, z) and the number of atoms (N) of the <111> STGBs models in the simulation of mechanical properties.

θ (°)	Σ	x (Å)	y (Å)	z (Å)	N
0.00	1	47.93	86.09	45.19	36612
7.34	61	56.10	97.17	52.72	55948
9.43	37	40.20	92.85	52.72	38316
11.64	73	59.30	68.47	52.72	41681
13.17	19	48.81	84.53	45.19	36278
15.18	43	38.40	99.76	52.72	39369
17.90	31	66.51	96.00	37.65	46844
21.79	7	56.36	97.61	37.65	40374
24.43	67	51.35	59.30	60.24	35730
27.80	13	74.55	86.09	37.65	47033
30.59	97	46.42	93.81	45.19	38327
32.20	13	64.78	74.80	45.19	42661
38.21	7	50.79	87.99	45.19	39321
42.10	31	55.34	85.20	52.72	48529
43.57	49	47.93	86.09	45.19	36612
46.83	19	56.10	97.17	52.72	55948
50.57	37	40.20	92.85	52.72	38316
53.99	91	59.30	68.47	52.72	41681
60.00	3	48.81	84.53	45.19	36278

Table S7. The misorientation angle (θ), Σ value, the dimensions of the simulation box (x, y, z) and the number of atoms (N) of the <100> STGBs models in the simulation of irradiation.

θ (°)	Σ	x (Å)	y (Å)	z (Å)	N
0.00	1	156.52	313.05	156.52	746496
9.53	145	157.07	314.14	156.52	751068
12.68	41	157.49	314.98	156.52	755244
16.26	25	153.72	307.45	156.52	720108
18.92	37	158.68	317.37	156.52	767160
22.62	13	155.19	310.38	156.52	734004
25.06	85	160.34	320.69	156.52	782784
28.07	17	161.34	322.69	156.52	793152
30.51	65	148.72	297.44	156.52	673452
33.40	109	158.88	317.76	156.52	768600
36.87	5	164.99	329.98	156.52	829368
39.97	137	152.67	305.35	156.52	709740
43.60	29	163.90	327.80	156.52	818496
46.40	29	165.56	331.13	156.52	835308
53.13	5	155.55	311.111	156.52	737280
59.49	65	157.74	315.49	156.52	758160
61.93	17	152.11	304.23	156.52	705348
64.01	89	164.07	328.15	156.52	819468
67.38	13	156.76	313.53	156.52	748692
71.08	37	149.61	299.22	156.52	682020
73.74	25	152.18	304.36	156.52	705600
77.32	41	167.04	334.08	156.52	850176
79.61	61	169.79	339.58	156.52	878220
82.37	113	161.76	323.53	156.52	796824
90.00	1	159.87	319.74	156.52	780624

Table S8. The misorientation angle (θ), Σ value, the dimensions of the simulation box (x, y, z) and the number of atoms (N) of the <110> STGBs models in the simulation of irradiation.

θ (°)	Σ	x (Å)	y (Å)	z (Å)	N
0.00	1	156.53	307.45	159.87	748800
7.35	243	143.78	338.89	159.87	756564
13.44	73	157.61	297.19	159.87	727520
20.05	33	141.29	299.73	159.87	658068
26.53	19	160.82	303.24	159.87	757147
31.59	27	135.56	319.51	159.87	672572
38.94	9	147.58	313.06	159.87	718725
44.00	57	164.13	324.97	159.87	828804
50.48	11	144.21	326.30	159.87	732095
55.88	41	157.49	334.09	159.87	816985
61.02	97	151.40	299.76	159.87	704587
70.53	3	159.76	331.36	159.87	823082
77.88	81	156.53	332.05	159.87	807011
80.63	43	171.07	322.57	159.87	857211
86.63	17	143.42	304.24	159.87	678823
90.58	99	173.05	305.91	159.87	822004
93.37	17	152.12	322.69	159.87	762442
99.37	43	161.29	285.12	159.87	714219
109.47	3	150.62	340.81	159.87	798396
117.56	67	151.00	320.31	159.87	751237
121.00	33	158.95	349.68	159.87	863891
124.12	41	167.04	314.98	159.87	817305
129.52	11	163.15	346.10	159.87	876633
136.00	57	162.48	328.27	159.87	828457
141.06	9	156.53	332.05	159.87	808414
145.11	89	145.02	328.15	159.87	738875
148.41	27	159.76	316.30	159.87	785119
153.47	19	151.62	321.63	159.87	758664
157.15	51	153.69	310.51	159.87	741470
159.95	33	149.86	353.23	159.87	822346
163.90	51	155.25	351.30	159.87	847506
180.00	1	156.53	332.05	159.87	808414

Table S9. The misorientation angle (θ), Σ value, the dimensions of the simulation box (x, y, z) and the number of atoms (N) of the <111> STGBs models in the simulation of irradiation.

θ (°)	Σ	x (Å)	y (Å)	z (Å)	N
0.00	1	159.76	319.75	150.62	750000
7.34	61	144.08	332.73	150.62	702703
9.43	37	168.31	323.92	150.62	799132
11.64	73	181.99	315.22	150.62	840963
13.17	19	160.82	324.97	150.62	766041
15.18	43	174.60	322.57	150.62	825692
17.90	31	148.25	342.36	150.62	743967
21.79	7	146.42	338.14	150.62	725486
24.43	67	174.35	352.32	150.62	900502
27.80	13	153.60	354.73	150.62	798897
30.59	97	157.34	363.36	150.62	837976
32.20	13	166.28	345.60	150.62	842396
38.21	7	154.98	325.37	150.62	739384
42.10	31	154.06	355.79	150.62	803483
43.57	49	149.11	344.34	150.62	752436
46.83	19	162.48	321.63	150.62	766149
50.57	37	161.96	336.63	150.62	799261
53.99	91	152.40	351.95	150.62	786249
60.00	3	166.02	340.81	150.62	829081
